# Characterization and performance of the Allegro™ STR 1000 single-use stirred tank bioreactor system

**DOI:** 10.1186/1753-6561-9-S9-P60

**Published:** 2015-12-14

**Authors:** Kerem Irfan, Byron Rees, Tim Barrett, Bojan Markicevic, Camille Segarra

**Affiliations:** 1 Pall Corporation, 5 Harbourgate Business Park, Southampton Road, Portsmouth, PO6 4BQ, UK

## Background

The benefits of single-use technologies in both upstream and downstream operations are now widely acknowledged by the biopharmaceutical industry, and have resulted in radical changes to the design and operation of many pharmaceutical processes. In mammalian cell culture, multi-use, cleanable glass or stainless steel stirred tank reactors (STRs) have been used successfully for growth of suspended cell lines for both small and large scale systems. However, to achieve the same or better performance from a single-use bioreactor presents a significant challenge to product designers and developers.

The poster describes the studies aimed at characterizing the fluid dynamics of the Allegro STR 1000 bioreactor by measurement and modelling of specific physical parameters known to be critical for mammalian cell culture: more specifically, oxygen transfer rates, mixing times and carbon dioxide stripping rate.

## Materials and methods

The study was conducted to determine the mixing efficiency, kLa O2 and CO2evacuation rate. For the mixing study, pH probes were located in eight different locations. For kLa O2 and CO2 evacuation rate determination two probes in the standard probe inserts were used (P1 & P2). A cell culture media simulant was used for all experiments: bicarbonate buffer, pluronic F68, antifoam A.

## Results

Mixing studies were conducted by performing a single shot addition of 4M NaOH and then measuring the change in pH of 1000 L of cell culture media simulant at ambient temperature. No significant difference was seen in the mixing time measured between the eight probe positions. Mixing time was defined as the time required to reach 100 ± 5 % of the final pH value. 1000 L of media simulant was heated up to 37 ºC and brought down to pH 6.5 using CO2 gas and maintained stable for a duration of 2 minutes. A pCO2 probe recorded the initial pCO2. Air was then sparged through the ring sparger of the bioreactor at the desired flow rate and the change in pH measured.

## Conclusions

Our studies have shown that, the Allegro STR 1000 single-use bioreactor can be engineered suitably to provide a practical alternative to stainless steel bioreactors. By evaluating the hydrodynamics of the Allegro STR 1000bioreactor, we established that the direct drive large impeller could provide power input for fluid volumes up to 1000 L that can support the growth of mammalian cells. This power transfer in conjunction with a customized cubical biocontainer with integrated baffles produced homogeneous mixing. User experience is also enhanced by providing features that make the installation and use of the bioreactor intuitive and easy.

**Figure 1:**
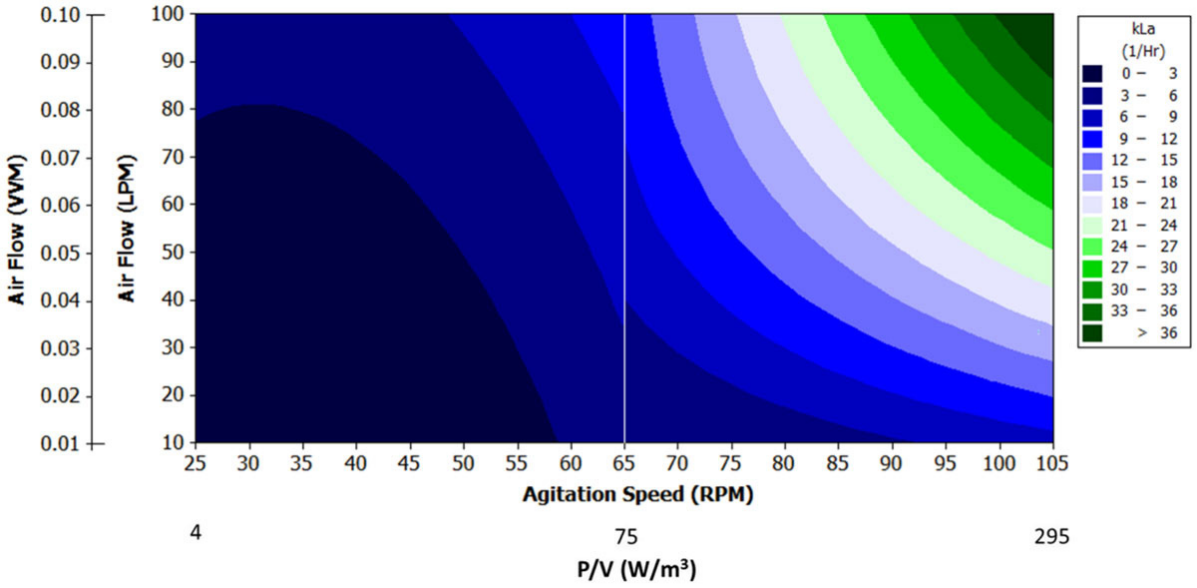
**Contour plots for kLa_O2_2080 values at various impeller agitation speeds and airflow rates**. Two DOE models required to capture change in curvature with RPM (25 to 65 RPM & 65 to 105 RPM)

